# Impact of Pulsed Electric Fields Combined with Dissolved Oxygen and Ferrous Ions on the Aroma and Components of Strong-Flavor Baijiu

**DOI:** 10.3390/foods14071097

**Published:** 2025-03-21

**Authors:** Jin Lu, Zhilei Zhou, Mengyang Huang, Zhongwei Ji, Hui Qin, Jian Mao

**Affiliations:** 1 School of Food Science and Technology, National Engineering Research Center of Cereal Fermentation and Food Biomanufacturing, Jiangnan University, Wuxi 214122, China; lujinyouxiang@163.com (J.L.); zhouzl1985@126.com (Z.Z.); 2 National Engineering Research Center of Solid-State Brewing, Luzhou 646000, China; hmy@163.com (M.H.); qinhui@163.com (H.Q.); 3 Jiangnan University (Shaoxing) Industrial Technology Research Institute, Shaoxing 312000, China; 4 National Engineering Research Center for Huangjiu, Shaoxing 312000, China

**Keywords:** strong-flavor baijiu, pulsed electric field, dissolved oxygen, ferrous iron ion, sensory evaluation, volatile organic compounds, multivariate statistical analysis

## Abstract

This research examined the influences of electric field strength and pulse frequency of pulsed electric field (PEF) treatment, along with the combined effects of dissolved oxygen and ferrous iron ions on the aroma and components of strong-flavor baijiu. PEF treatment improved fruity aromas and decreased the pit mud odor. Electric field strength promoted the production of short-chain fatty acid esters, while pulse frequency facilitated the formation of acetal oxidation products. The most notable changes were observed at an electric field strength of 25 kV, and a pulse frequency of 350 Hz. Increasing dissolved oxygen significantly improves fruity and mellow aromas and promotes the generation of 17 compounds including ethyl lactate, ethyl butyrate, hexan-1-ol, octanoic acid, and 3-methylbutanal, while Fe^2^⁺ treatment reduces the fruity aroma of baijiu and significantly suppresses the production of 15 esters including ethyl hexanoate, hexyl hexanoate, and ethyl lactate. Dissolved oxygen may contribute to the generation of hydroxyl radicals and regulated oxidation reactions partially in baijiu. And, Fe^2+^ may react with organic acids to promote the hydrolysis of ester compounds. This study aims to offer valuable insights into the practical application of PEF in the flavor regulation of baijiu.

## 1. Introduction

Baijiu is a unique alcoholic beverage from China, and freshly distilled baijiu often exhibits undesirable sensory attributes, including pungency, harshness, immaturity, and unpleasantness, which require an extended aging period [1]. Aging is crucial for removing off-odors and integrating various flavor components, leading to a more balanced and harmonious aroma [1,2]. During the aging of baijiu, several physicochemical processes are considered essential for flavor development, such as hydrogen bonding, esterification, hydrolysis, redox reactions, and the Maillard reaction [3]. However, the natural aging process is slow, resulting in challenges such as low production efficiency and high costs. To address these limitations, several studies have employed strategies such as electrochemistry, electric fields, electromagnetism, microwaves, and irradiation to enhance flavor and investigate aging mechanisms, offering promising solutions for reducing time and space costs [4,5,6,7,8].

Pulsed electric field (PEF) technology, a type of electric field technology, is primarily applied in the wine and vinegar industries, with limited use in baijiu [9,10]. Compared to other technologies, it can change the physical and chemical properties of food while avoiding the destruction of flavored substances. Liang et al. highlighted the significant potential of PEF in winemaking, where it effectively improves flavor and phenolic content in wine [11]. Artemis K. identified 10 new volatile compounds in Xinomavro red wine using headspace solid-phase micro-extraction (HSPME) and gas chromatography-mass spectrometry (GC-MS) following PEF treatment with wood chips, which notably enhanced floral, fruity, and overall aromas [12]. The electric field strength and pulse frequency in PEF are critical factors affecting process quality and stability [6,13]. Increasing the electric field strength (15–25 kV/cm) in an ethanol–lactic acid model system enhanced esterification between lactic acid and ethanol, resulting in a 1.1-fold increase in ethyl lactate yield [14]. Additionally, increasing treatment intensity can alter pH levels, significantly improving the chelation of ferrous ions, copper ions, and amino acids such as glycine [15]. Furthermore, the solution characteristics and redox microenvironment must be systematically evaluated as critical factors in PEF effects. And, the oxidation process driven by oxygen is crucial for the formation of flavor compounds [16,17]. Free radicals are recognized as key initiators in the redox chemistry of wine [18,19]. The radicals produced by the electric field can initiate chain reactions, leading to the oxidative degradation of specific compounds. Hydroxyl radicals are the most produced ones [20]. During the natural aging of baijiu, hydroxyl radicals are mainly generated through the Fenton reaction (i.e., H_2_O_2_ reacts to produce ·OH under the catalysis of Fe^2+^). The highly oxidizing nature of ·OH enables it to react with carbon–carbon unsaturated bonds and aromatic rings in organic molecules through addition, substitution, electron transfer, and other reactions [21,22]. Limited research has been conducted on the effect of oxygen in PEF-processed baijiu systems, which may yield significant impacts on flavor.

This study aims to investigate the impact of PEF on the flavor properties of strong-flavor baijiu. The objectives of this work are (1) to assess the effects of electric field strength and pulse frequency on the electrical conductivity, dissolved oxygen concentration, and flavor compounds of strong-flavor baijiu; (2) to explore the role and mechanism of dissolved oxygen and transition metals in combination with PEF in enhancing the aging efficiency of baijiu. The results of this study are anticipated to provide valuable insights into the practical application of PEF in baijiu production.

## 2. Materials and Methods

### 2.1. Materials and Chemicals

The strong-flavor baijiu utilized in this research is a new distilled baijiu, produced by Luzhoulaojiao Co., Ltd. (Luzhou, China) in December 2023. All the baijiu samples were sealed and stored at 4 °C prior to treatment.

Sodium chloride was sourced from Sinopharm Chemical Reagent Co., Ltd. (Shanghai, China). Internal standards, including tertiary amyl alcohol, pentyl acetate, and 2-octanol, were purchased from Aladdin Reagent Co., Ltd. (Shanghai, China). A C_4_–C_25_ n-alkane mixture (Sigma-Aldrich, Shanghai, China) was employed to determine linear retention indices (RIs). A standard stock solution of Fe (PerkinElmer Instrument Co., Ltd., San Jose, CA, USA) was used. The following reference compounds for qualitative and quantitative analysis were acquired from Sigma-Aldrich Chemical Co., Ltd. (Shanghai, China): acetic acid (99%), acetal (≥98%), butyric acid (99%), butyraldehyde (≥99%), benzaldehyde (≥99%), benzene acetaldehyde diethyl acetal (97%), ethyl acetate (99.5%), ethyl propionate (99%), ethyl butyrate (99%), ethyl myristate (99%), ethyl valerate (99%), ethyl caprate (99%), ethyl isovalerate, ethyl heptanoate (99%), (98%), ethyl caprylate (99%), ethyl lactate (99%), ethyl palmitate (99%), furfural (99%), geranyl acetone (99%), hexanal (98%), hexyl hexanoate (99%), hexan-1-ol (99%), isobutyric acid (99%), isobutyraldehyde (≥99%), isovaleraldehyde (97%), isobutanol (≥97%), isoamyl alcohol (≥98%), lactic acid (99%), methanol (99%), octanoic acid (99%),pentyl hexanoate (99%), propyl n-octanoate (99%), p-cresol (98%), pentan-1-ol (99%), 1,1-diethoxydecane (97%), 1,1-diethoxynonane (97%), 1-propanol (99%), 2,3-butanedione (97%), 2,3-pentanedione (97%) 2-heptanone (99%), 2-octanone (≥98%), 2-butanone (≥99%), 2-pentanone (≥98%), 3-methyl-1-butanol (99%), and 3-methylbutyl pentanoate (99%).

### 2.2. PEF Treatment

#### 2.2.1. PEF System

**Figure A1:**
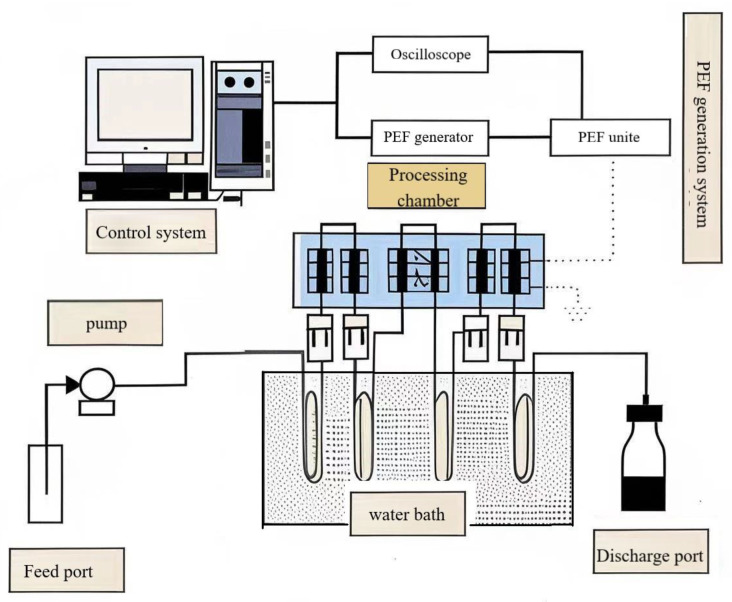
Simplified diagram of the PEF processing system.

A bench-scale PEF continuous system (OSU-4L, The Ohio State University, Columbus, OH, USA) with square-wave pulsed was used in this study [23]. The PEF system schematic diagram is shown in Figure A1. Throughout all the experiments, the temperature rise after treatment did not exceed 3 °C.

#### 2.2.2. Parameter Optimization

In the parameter optimization experiment, the baijiu was directly subjected to PEF treatment with different electric field strengths and pulse frequencies. The control group (referred to as CK) was not exposed to any PEF treatment, and the experimental groups are S1–S3 and F1–F3. According to the preliminary experiment, for the S1 to S3, the electric field strength was 15 kV, 25 kV, and 35 kV and the pulse frequency was 350 Hz; for the P1 to P3, the pulse frequency was 200 Hz, 350 Hz and 500 Hz, and the electric field strength was 25 kV.

#### 2.2.3. Experiment Design of Baijiu Sample Pretreated

The control group (referred to as G-PEF) was treated directly with PEF without any additives. Two groups of baijiu samples required pretreatment. One group underwent pretreatment using a micro-oxygenation (MOX) operation (referred to as G-PEF+DO) before the PEF treatment. The micro-oxygenation technique used in this study was based on Pérez-Magarin [22] with some modifications. The MOX process was conducted using a microporous diffuser, specifically the VinO_2_ 4-exit impact model, connected to 1 L glass bottles (225 mm in height and 101 mm in diameter). Oxygen was injected through a pressure-reducing valve and mass flowmeter, maintaining a dissolved oxygen concentration of 8.19 mg/L for 5 min while ensuring controlled airflow to prevent excessive bubbles (flow rate maintained below 1 mL/min). The other group (referred to as G-PEF+Fe^2+^) was treated with a ferrous iron ion buffer before the PEF treatment. A 200 μL ferrous iron ion solution (10.79 mmol/L) was added to a 100 mL baijiu sample. Finally, both the experimental and control groups were sampled following the unified PEF treatment (parameters defined in Section 2.2.2).

### 2.3. Sensory Analysis

#### 2.3.1. Panelists

The sensory evaluation panel consisted of twelve trained students (five males and seven females) from Jiangnan University, along with five baijiu tasters. The panelists were selected according to the national standard GB/T14195–93 [24]. The students involved were selected based on interest, health status (without alcohol addiction, alcohol exclusion, and alcohol allergy), availability for the entire study, and familiarity with distilled beverages using an initial recruitment questionnaire. In addition, assessors were required to complete sensory ability tests for basic taste identification and intensity ranking according to the guidelines of the ISO 8586:2012 standard [25]. The assessors were selected if they achieved at least 80% acuity on the sensory ability tests. They were provided with written informed consent and were paid for their participation. After qualifying for the assessment, the panelists underwent two months of sensory training (1 h for each) before participating in the evaluations. All the tests were conducted in a sensory evaluation laboratory at a temperature of 20 ± 1 °C. The sensory sessions received approval from the Jiangnan University Medical Ethics Committee (JNU202312IRB13).

#### 2.3.2. Sample Evaluation

Based on references and sensory descriptors proposed by the selected panel [24,26], eight odor descriptors associated with the sensory attributes of strong-flavor baijiu were identified: overall aroma intensity, cellar aroma, grain aroma, mellowness, fruity, pungency, sourness, and mud aroma. The panelists were asked to rate the intensity of each attribute on a ten-point scale, ranging from 0 to 9, where 0 indicated no odor and 9 represented a very strong odor.

### 2.4. Analysis of Volatile Compounds

The volatile compounds in the baijiu samples were analyzed using an Agilent GC-MS (7890A-5975C, Agilent Technologies, Santa Clara, CA, USA) and an Agilent GC-FID (8890C, Agilent Technologies, Santa Clara, CA, USA). A diluted baijiu sample (6.0 mL) with a final ethanol content of 8 %, vol was mixed with 10 µL of internal standard solution (tertiary amyl alcohol, 8.05 g/L; pentyl acetate, 10.33 g/L; and 2-octanol, 100 mg/L) [27]. GC separation was performed on a DB-FFAP capillary column (60 m × 0.25 mm × 0.25 µm; J&W Scientific, San Jose, CA, USA) with a flow rate of 1.2 mL/min. The oven temperature was initially set at 40 °C and held for 1 min, then raised to 100 °C at 2.5 °C/min and held for 1 min. Then, the temperature increased to 160 °C at 3 °C/min without holding, and finally rose to 230 °C at 5 °C/min, where it was held for 20 min. A PAL 3 autosampler (CTC Analytics AG, Zwingen, Switzerland) with an SPME Fiber (80 μm thickness, 10 mm length, DVB/C-WR/PDMS) (Agilent Technology Co., Ltd., Santa Clara, CA, USA) was used to extract volatile compounds from the sample’s headspace. The sample was preheated at 60 °C for 5 min and extracted at 60 °C for 40 min. The mass selective detector was configured as follows: the interface temperature was 280 °C, the quadrupole temperature was set to 150 °C, and the ion source temperature was maintained at 230 °C. Electron impact (EI) ionization was performed at 70 eV, and a full scan (*m*/*z* 30–350) was conducted. The GC-FID oven temperature program followed the same steps as the GC-MS analysis. Both the injector and FID temperatures were set to 250 °C.

Qualitative identification was carried out by comparing the retention times and retention indices (RIs) of the target compounds with those of reference standards. The RIs were determined using retention times of homologous n-alkanes (C_4_–C_25_). The concentrations of volatile organic compounds (VOCs) were calculated by dividing the peak area of the target compound by the peak area of the internal standard.

### 2.5. Alcoholic Strength, Electrical Potential, pH, DO, and Conductivity Analysis

Alcoholic strength was measured following the GB 10345–2022 standard [28]. Electrical potential, pH, and conductivity were assessed using a multi-parameter tester (Multi 3630, WTW, BY, Munich, Germany). Dissolved oxygen (DO) levels were measured in accordance with GBT 7489–1987 using a portable DO meter (S9-Field kit, Mettler Toledo, Columbus, OH, USA).

### 2.6. ICP-MS Analysis

An 8-fold dilution of baijiu was analyzed for iron content by inductively coupled plasma mass spectrometry (ICP-MS, Thermo Fisher Scientific Inc., Waltham, MA, USA). The instrument parameters included RF power at 1.50 kW, plasma flow at 15.0 L/min, auxiliary flow at 1.0 L/min, and nebulizer flow at 0.10 L/min.

### 2.7. Electron Paramagnetic Resonance (EPR) Spin Trapping

Aqueous sample solutions (1 mg/mL) were prepared by ultrasonication for 15 min at 25 °C. Subsequently, 200 μL aliquots were mixed with equal volumes of 100 mM DMPO solution through vortex agitation (30 s, 2000 rpm). The resulting mixtures were immediately transferred into quartz capillaries. EPR spectra (EMXplus-10/12, Bruker, Bremen, Germany) were recorded at room temperature, and spin adducts were quantified. The sweep width was set to 200.00 G, with microwave power at 19.45 mW. The modulation frequency and amplitude were set to 9.85 GHz and 1.00 G, respectively.

### 2.8. Statistical Analysis

The results are presented as the mean ± standard deviation (*n* = 3). The data were processed and plotted using the Origin 2018 software (Origin Lab Co., Northampton, MA, USA) and Microsoft Office Excel 2019 (Microsoft Co., Ltd., Redmond, WA, USA). For analysis, the processed data were evaluated using the Simca 13.0 and SPSS 25 software (SPSS Inc., Chicago, IL, USA), one-way analysis of variance (ANOVA), and Duncan’s multiple range tests were carried out with the SPSS 25.0 software to evaluate significant differences in the assay (*p* < 0.05). Statistical analysis was performed using Origin 2018. Substantial differences were visualized using the Simca 13.0 and online websites (https://www.omicstudio.cn (accessed on 15 December 2024)).

## 3. Results

### 3.1. Effects of PEF Treatment on Physicochemical Parameters of the Strong-Flavor Baijiu

The physicochemical parameters of strong-flavor baijiu before and after the PEF treatment are summarized in Table 1. The alcohol content exhibited comparable decreasing trends, while conductivity and dissolved oxygen (DO) showed a notable increase after treatment (*p* < 0.05). This increase is most likely attributed to the heightened collision frequency of active molecules or ions induced by the PEF treatment. Ethanol and water molecules in the solution are excited or ionized, generating charged particles and active oxygen [29,30]. The analysis of various PEF processing parameters revealed significant differences in conductivity and DO post-treatment, with the intensity of these effects varying based on the strength of the treatment. The F1 baijiu sample showed the highest conductivity (17 μs/cm), significantly higher than the other samples (*p* < 0.05). Gulsum also indicated that the conductivity of wine increased significantly after PEF treatment [31]. There was no significant difference in DO treatment with high electric strength (25–35 kV/cm). This is probably because PEF is a short-duration process with limited impact on DO in a short time.

### 3.2. Effect of PEF Treatment on Baijiu Flavor Perception

**Figure 1:**
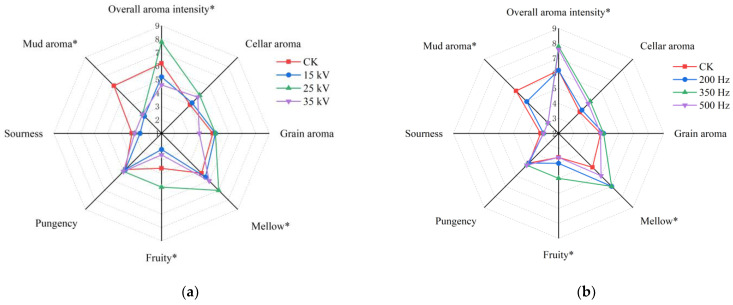
(**a**) Aroma profiles of strong-flavor baijiu under different electric field strengths; (**b**) aroma profiles of strong-flavor baijiu at different pulse frequencies. Significance is indicated at * (*p* < 0.05).

The baijiu samples were blindly evaluated by a sensory panel using eight odor descriptors to assess the overall aroma. The final results are displayed as a radar chart in Figure 1. Significant changes were observed in the overall aroma intensity, fruity aroma, and mud aroma, with the mud aroma decreasing notably. When the pulse frequency was 350 Hz, and the electric field strength was 25 kV/cm; the highest scores were recorded for overall aroma intensity and fruity aroma, while the overall aroma intensity was significantly lower compared to the CK group when the strength was increased or decreased. Furthermore, when the strength was maintained at 25 kV/cm, and the pulse frequency was increased from 200 Hz to 500 Hz, the overall aroma intensity, fruity aroma, and mellow scores improved significantly. Previous studies have shown that PEF treatment can enhance the fruit aroma in wine [11,12].

In general, modifications in electric field strength tend to have a greater effect on the flavor of baijiu, with excessively high field strength possibly leading to a loss of overall aroma. In contrast, the influence of pulse frequency changes on aroma appears less pronounced. Overall, when the electric field strength was set at 25 kV, and the pulse frequency at 350 Hz, both the overall aroma intensity and fruity aroma were significantly enhanced, while the mud aroma was reduced.

### 3.3. Effect of Pulse Parameters on the Flavor Compounds of Strong-Flavor Baijiu

#### 3.3.1. Effect of Electric Field Strength and Pulse Frequency on Flavor of Strong-Flavor Baijiu

The water and ethanol in baijiu constitute over 98%, with the remaining less than 2% comprising organic compounds such as esters and acids. These trace compounds are critical in determining the style and quality of baijiu. In this study, 125 volatile compounds were identified, including 70 esters, 13 aldehydes and ketones, 19 acids, 15 alcohols, and 8 benzene derivatives (the details are shown in Table A1). The impact of electric field strength and pulse frequency on tare concentrations of various volatile flavor compounds in baijiu is presented in Table 2. Significant differences were observed in the concentrations of the five main compounds. Following treatment, the contents of alcohols, aldehydes, ketones, aromatics, and acids in baijiu were significantly higher than those in the CK group (*p* < 0.05), indicating the effect of PEF. Overall, as the pulse frequency increased from 200 Hz to 500 Hz, the total concentration of flavor compounds in baijiu increased by 24.74%. Similarly, the ester concentration in baijiu followed this trend, increasing from 2707 mg/L (CK) to 3897 mg/L (500 Hz, F3). Furthermore, recent investigations have demonstrated that electric field treatment significantly enhances ester [12,32]. However, the ester concentration was not significantly affected by variations in electric field strength. In all the baijiu samples, esters constituted the majority of the volatile components, which are primarily formed during fermentation and aging processes, contributing strong fruity and floral aromas. The increased ester content enhances the potential for a stronger fruity aroma in baijiu.

**Figure 2:**
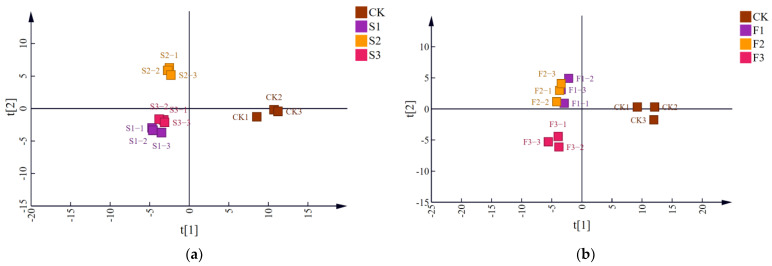
(**a**) The score plot of Strong-flavor baijiu samples with different pulse frequencies of PEF based on all compounds; (**b**) the score plot of baijiu samples with different electric field strengths of PEF. S1~S3 and F1~F3 were the experimental groups, and the electric field strength from S1 to S3 was 15 kV, 25 kV, and 35 kV. The pulse frequency from F1 to F3 was 200 Hz, 350 Hz, and 500 Hz.

**Figure 3:**
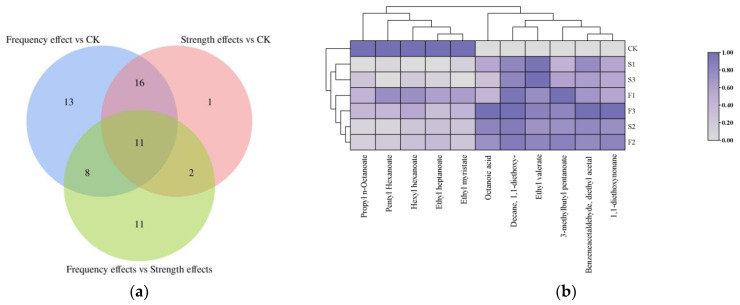
(**a**) Venn plots of differential compounds for different comparison groups; (**b**) Heat map of common differential compounds. (CK was the control group. S1–S3 and F1–F3 were the experimental groups, and the electric field strength from S1 to S3 was 15 kV, 25 kV, and 35 kV, and the pulse frequency was 350 Hz. The pulse frequency from F1 to F3 was 200 Hz, 350 Hz, and 500 Hz, and the electric field strength was 25 kV.)

To facilitate analysis, samples with the same parameters at different treatment levels were considered as intra-group samples. Partial Least Squares Discriminant Analysis (PLS-DA) was performed to examine inter-group and intra-group variability. Following dimensionality reduction, relative coordinate points for each principal component were established, resulting in a score chart (Figure 2). The CK group and PEF-treated groups were clearly distinguishable, with the group treated at 25 kV or a pulse frequency of 500 Hz showing greater inter-group distinction than other experimental groups. To identify the main compounds affected by electric field strength and pulse frequency, PLS-DA and ANOVA were employed, and the number of differential compounds with VIP > 1 and *p* < 0.05 among groups is presented in Figure 3a.

According to the Venn diagram results (Figure 3a), 11 common differential compounds were determined which were identified as the most capable of explaining the influence of the PEF treatment (Figure 3b). The concentrations of esters such as pentyl hexanoate, propyl n-octanoate, hexyl hexanoate, and ethyl myristate decreased significantly after the PEF treatment. These esters, primarily long-chain fatty acid esters (LCFAEEs, C > 10), were notably reduced which may be due to PEF action to promote the occurrence of ester hydrolysis [33]. Additionally, this may be attributed to the alteration in the surface charge distribution of ester molecules following the PEF treatment, which enhances their ability to interact with water and thereby facilitates the hydrolysis of long-chain fatty acid esters [34]. Additionally, ester formation is a dynamic equilibrium, and numerous studies have demonstrated that electric field treatments and related interventions can enhance esterification in fermented beverages [14,32,35,36]. Ethyl valerate and 3-methyl butyl pentanoate, which are characterized by fruity aromas, showed significantly increased levels after PEF treatment [37,38]. Sensory experiments revealed that fruity aromas increased post-treatment. In addition, octanoic acid and 3-methylbutyl pentanoate contents were highest at low pulse frequencies. The increase in octanoic acid may result from alcohol oxidation and ester hydrolysis during early aging. In addition, there have been studies indicating that the octanoic acid content in baijiu showed an increasing trend after electric field treatment [32,39]. Additionally, acetals in baijiu, such as benzene acetaldehyde diethyl acetal (also known as Phenylacetaldehyde Diethyl Acetal) and 1,1-diethoxynonane (also known as 1,1-Diethoxy-n-nonane), also increased. As acetals arise from alcohol and aldehyde condensation and typically exhibit a pleasant aroma, the increase in their content suggests that electric field reactions may facilitate oxidation reactions leading to aldehyde formation.

#### 3.3.2. Correlation Analysis of Electric Field Strength, Pulse Frequency, and Differential Compound

**Figure 4:**
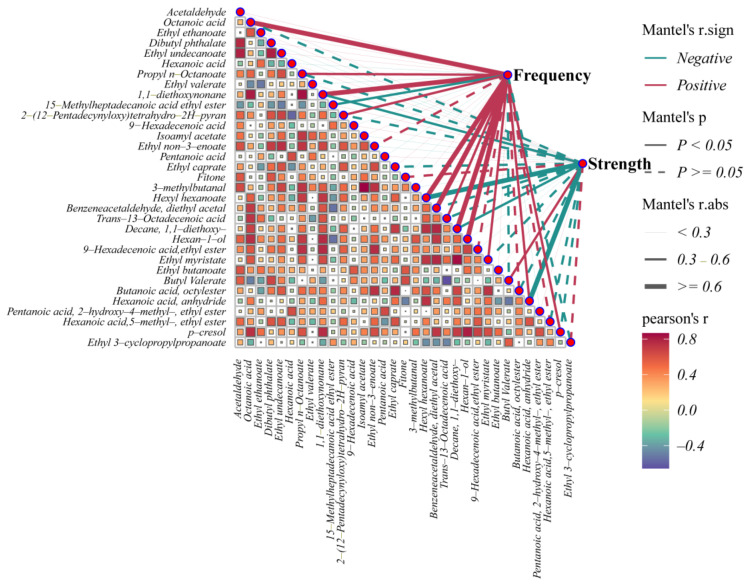
Correlation analysis between flavor substances and electric strength, pulse frequency in strong-flavor baijiu treated in various PEFs (red indicates a positive correlation and blue indicates a negative correlation; solid line indicates *p* < 0.05). The correlations were determined by the Pearson correlation analysis and Mante test (For interpretation of the details in this figure, the reader is referred to Table A2 of Appendix B).

Through PLS-DA of the inter-group comparison results, a total of 33 compounds were screened for further analysis to identify the main substances influenced by different parameters (Figure 4). Using Pearson correlation calculations, hexyl hexanoate, and hexanoic acid, anhydride exhibited significant negative correlations with electric field strength. At the same time, hexyl hexanoate had correlation coefficients exceeding 0.6 and displayed positive correlations with pulse frequency. Additionally, butyl valerate was positively correlated with increasing electric field strength (*p* < 0.05). Hexyl hexanoate is a primary flavor compound in baijiu, the hexyl hexanoate content decreased significantly after the PEF treatment. Notably, no significant differences in hexyl hexanoate levels were detected across the varying electric field intensities. However, when maintaining a constant electric field strength of 25 kV, the hexyl hexanoate content exhibited a declining trend with increasing pulse frequency. These results may indicate that hexyl hexanoate demonstrates higher sensitivity to electric field strength modifications compared to pulse frequency adjustments. Additionally, the contents of 1,1-diethoxynonane and 1,1-diethoxydecane showed a negative correlation with electric field strength. Acetals in baijiu are formed through the condensation of alcohol and aldehydes and generally possess a pleasant aroma [40].

Octanoic acid, propyl n-octanoate, 2-(12-pentadecynyloxy) tetrahydro-2H-pyran, benzene acetaldehyde diethyl acetal, trans-13-octadecenoic acid, hexan-1-ol (1-hexanol), ethyl myristate, hexanoic acid, anhydride, p-cresol, and other compounds exhibited significant positive correlations with pulse frequency. In contrast, 15-methylheptadecanoic acid ethyl ester showed a significant negative correlation with pulse frequency. Among these, the correlation coefficients of octanoic acid, 1,1-diethoxynonane, hexyl hexanoate, benzene acetaldehyde diethyl acetal, 1,1-diethoxydecane, and 1-hexanol were all greater than 0.6. Propyl caprylate and ethyl myristate are associated with an iris-like aroma [32]. These compounds exhibited positive correlations with pulse frequency. This observation supports the conclusion that variations in electric field strength strongly influence baijiu flavor [40].

PEF supplies the energy required to drive chemical reactions, facilitating a shift in the equilibrium between electronic transition, electro esterification, and hydrolysis [14,41]. Under the influence of the field energy, LCFAEEs undergo hydrolysis, making it easier to form SCFAEEs following electric field treatment. Consequently, an increase in electric field strength does not significantly enhance the oxidation reactions in baijiu. Instead, an increased number of unit discharges is more likely to promote oxidation reactions, resulting in the production of aldonic acids. Variations in pulse frequency and electric field strength influence the degree of oxidation and ester hydrolysis in baijiu to different extents. These changes also affect the generation of aging markers associated with fruity aromas in baijiu. The appropriate adjustment of electric field parameters offers a strategy for optimizing baijiu flavor.

### 3.4. Effect of Oxidant Combined with PEF on the Strong-Flavor Baijiu

**Figure 5:**
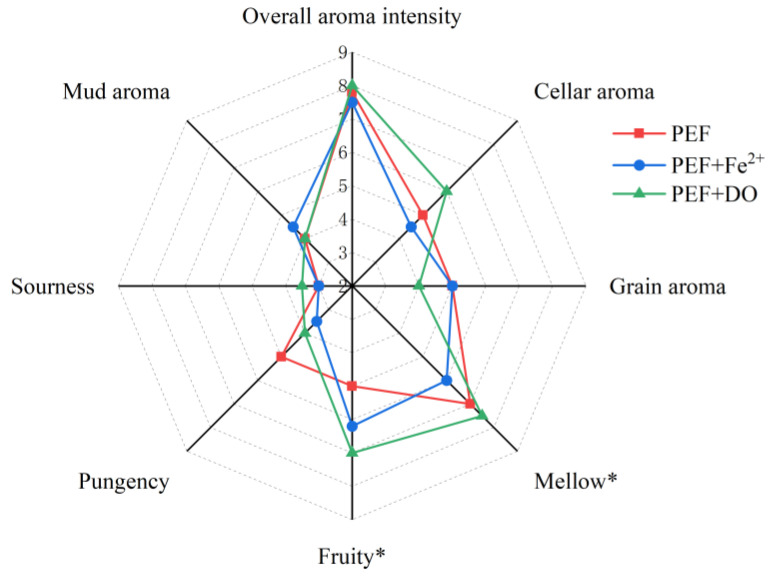
Aroma profiles of strong-flavor baijiu under various treatments. (* indicates *p* < 0.05).

Oxygen and metals play important roles in the storage process of baijiu [42]. In this study, the aroma and volatile flavor compounds were analyzed before and after the action of dissolved oxygen and ferrous ion-assisted PEF. As shown in Figure 5, after various PEF treatments, only the fruity and mellow aromas have significant differences. Increasing dissolved oxygen significantly improves fruity aroma, and a previous study also demonstrated that the controlled oxidative treatment of baijiu significantly weakens the raw aroma. In addition, the existence of Fe^2+^ weakens the mellow aroma of strong-flavor baijiu, and there is no significant difference in other aromas.

**Figure 6:**
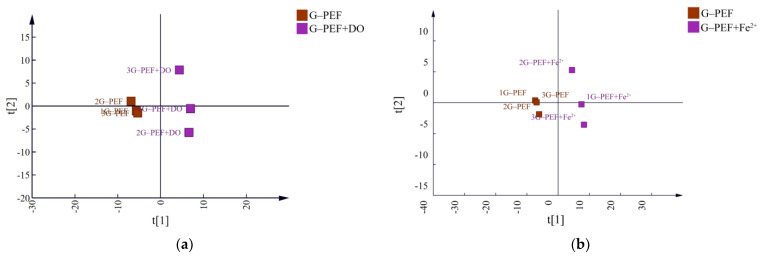

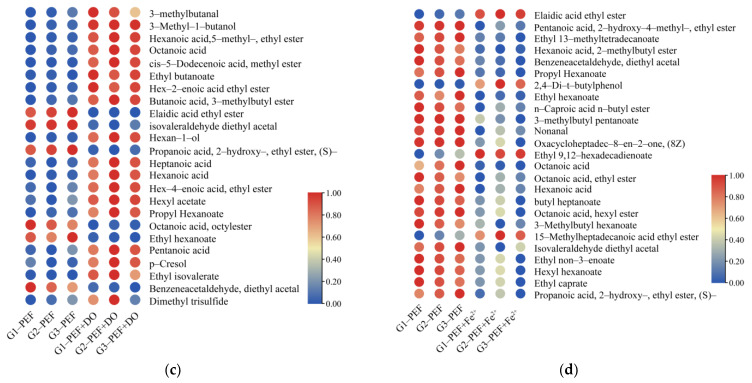
(**a**) Score chart of volatile components in strong-flavor baijiu after increasing dissolved oxygen. (**b**) Score chart of volatile components in strong-flavor baijiu after increasing Fe^2+^. (**c**) Heat maps of the main differential compounds for G-PEF and G-PEF+DO. (**d**) Heat maps of the main differential compounds for G-PEF and G-PEF+Fe^2+^.

Using HS-SPME combined with GC-MS to analyze volatile flavor compounds before and after treatment, the principal component analysis (PCA) results (Figure 6a,b) indicated a clear separation and significant differences among G-PEF+DO, G-PEF+Fe^2+^, and G-PEF groups. The PLS-DA model was utilized to select classification and discriminant variables. Differential compounds with VIP > 1 were identified (Figure 6c,d).

As shown in Figure 6c, a total of 24 differential compounds were identified in the dissolved oxygen treatment group, including 12 esters, 5 organic acids, 2 acetals, 1 aldehyde, 2 alcohols, 1 sulfur compound, and 1 aromatic compound. Among them, 17 compounds such as octanoic acid, heptanoic acid, hexanoic acid, pentanoic acid, and ethyl hexanoate showed a significant upward trend. The increased dissolved oxygen in raw baijiu elevated the contents of isoamyl alcohol, likely due to the hydrolysis promoted by the PEF. The increase in dissolved oxygen likely promotes oxidation, converting alcohols into corresponding acids and esters to increase ester concentration [21,43]. This leads to increased acid content like hexanoic, pentanoic, heptanoic, and octanoic acids, consistent with studies showing that electrochemical oxidation also increases acid content in baijiu and promotes ester formation [17]. Notably, since ester formation is reversible, oxidation favors ester production when ester concentrations are low, driving the reaction toward balance [2]. In addition, dissolved oxygen addition caused significant decreases in 6 compounds: elaidic acid ethyl ester, isovaleraldehyde diethyl acetal, ethyl hexanoate, ethyl lactate, phenylacetaldehyde diethyl acetal, and isovaleraldehyde diethyl acetal. Most of these esters are LCFAEEs, and their contents decrease under the action of PEF. The addition of dissolved oxygen may promote the hydrolysis of LCFAEEs. In addition, research by Wei et al. demonstrated that the dimethyl trisulfide described with salty and cooked onion flavors significantly increases during storage in pottery jars [44]. Studies have shown that dimethyl trisulfide can enhance the fruity aroma in baijiu through synergistic effects and serves as a key component in improving baijiu’s fruit aroma [45,46]. And, the content of dimethyl trisulfide also exhibits an increasing trend after the addition of dissolved oxygen.

In the ferrous ion treatment group, 25 differential compounds were detected, including 18 esters, 4 aldehyde/ketones, 2 acids, and 1 aromatic compound. The substances including elaidic acid ethyl ester, 2,4-Di-t-butylphenol, ethyl 9,12-hexadecadienoate, and 15-methylheptadecanoic acid ethyl ester showed an increasing trend, while 21 others significantly decreased. This might be because adding Fe^2+^ boosts ester hydrolysis, cutting ester levels. Also, metal ions could bind with acids and other aroma compounds to form metal–aroma compound complexes or colloidal particles [47]. Under pulsed electric fields, reactant collisions are increased, possibly disrupting the original reaction equilibrium. Additionally, the hexanol content decreased with increased dissolved oxygen and ferrous ions. This may occur because hexanol, with lower steric hindrance, is more susceptible to oxidation by ferrous ions. Both oxygen and ferrous ions significantly influence the oxidation system under PEF [2].

Notably, hexanoic and octanoic acids exhibited divergent responses to oxygen and ferrous ions. While oxygen and ferrous ions generally promote oxidation (converting alcohols to aldehydes and subsequently to acids), increased dissolved oxygen elevated these acids’ contents through enhanced oxidation. Conversely, higher ferrous ion levels reduced acid concentrations, likely through two mechanisms: (1) the formation of stable complexes with organic acids [43,47], and (2) the catalytic promotion of condensation reactions to form lactones and anhydrides [2]. Hexanoic and octanoic acids contribute to cellar aroma and mellow mouthfeel. Oxygen-assisted electric field treatment reduced alcohol content, increased aldehydes and acids, and stabilized these compounds during storage. As crucial precursors of esters, acids play a vital role in baijiu flavor development.

**Figure 7:**
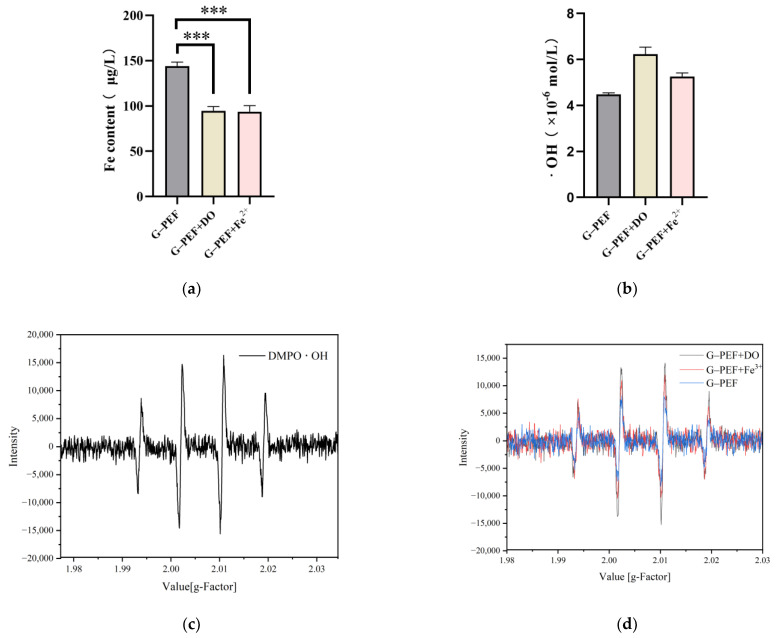
(**a**) shows the Fe element content before and after various treatments (*** indicates *p* < 0.01). (**b**) shows the content of hydroxyl radical after various treatments of the samples. (**c**) shows the EPR spectrum of •OH with DMPO. (**d**) shows the hydroxyl radical of samples with PEF combined with DO, Fe^2+^.

Because of production-related factors, raw baijiu contains certain amounts of iron. The determination of iron content in raw baijiu after the PEF treatment revealed a significant reduction in Fe levels (Figure 7a). This phenomenon could be attributed to the precipitation of iron ions formed through the reaction between Fe^2^⁺ and hydroxide ions (OH⁻) generated during Fenton-type processes. The formed iron hydroxide precipitates were subsequently removed during filtration [47,48,49].

Furthermore, the hydroxyl radicals in baijiu, both prior to and following the PEF treatment, were assessed using the electron spin resonance (ESR) technique. The formation of hydroxyl radicals was confirmed in the baijiu treated with PEF (Figure 7b). These radicals are sufficiently stable to be captured by nitrone spin traps (such as DMPO) and quantified by measuring the intensity of the ESR spectrum associated with the spin adduct (Figure 7c). The presence of these radicals is evident in the samples shown in Figure 6, indicating that pulsed electric field treatment induces free radicals in the baijiu system. As illustrated in Figure 7b, the intensity of hydroxyl radicals increased in the samples after PEF treatment compared to the G-PEF group, suggesting that dissolved oxygen and transition metals contribute, either directly or indirectly, to the formation of hydroxyl radicals [19]. In addition, the results showed that the content of hydroxyl radical produced by increasing the DO in the raw baijiu was higher than that of Fe^2+^, which may be caused by the precipitation of iron ions generated by the reaction with hydroxyl radical, resulting in the decrease in the content of Fe in the solution (Figure 7d). When PEF is applied, ionization of the solution generates O_3_, which possesses a strong oxidizing effect and may cause molecular oxygen in the solution to partially convert into reactive oxygen species. At higher pH levels, the conjugate base HO_2_– of H_2_O_2_ accelerates the decomposition of O_3_ and H_2_O_2_ [7,50]. Since •OH is the primary species responsible for the degradation of organic matter in these reactions, it is likely that the electric field action promotes the formation of hydroxyl radicals. During the oxidation process of raw baijiu, reactive oxygen species assist in accelerating the oxidation rate of alcohols.

In the PEF-treated samples, the influence of ferrous ions is notably more intense than that of DO. The understanding of baijiu’s spontaneous maturation in modern chemistry is largely centered on the oxidation process, with alcohols, phenols, and aldehydes as key substrates. The electric field assists in converting DO into reactive oxygen species, while the energy from the electric field facilitates electron transfer. Through this combined effect, compared to the single PEF treatment, oxidation reactions in the baijiu system are enhanced, leading to considerable changes in alcohol and aldehyde compounds. The control of oxygen and metals is crucial in pulsed electric field industrial applications.

## 4. Conclusions

In conclusion, this study investigated the effects of PEF treatment, baijiu systems affected by dissolved oxygen and ferrous iron, and their combined influence on the chemical composition and sensory characteristics of strong-flavor baijiu. PEF equipped with the potential to modulate the baijiu’s sensory attributes, can be employed as a processing technique to regulate and control the levels of volatile flavor compounds in strong-flavor baijiu. Comparatively, the intensity of pulse frequency is more likely to promote oxidation reactions, resulting in the production of aldehyde and acid. Based on the experimental data, the following pulse parameters were chosen for processing: a frequency of 350 Hz and an electric field strength of 25 kV. Increasing the dissolved oxygen in the baijiu system can promote the production of acids and esters, likely due to the generation of hydroxyl radicals that regulate oxidation processes. Ferrous ions significantly reduced ester concentrations while decreasing fruity aromas in raw baijiu. In practical applications, baijiu flavor can be adjusted by controlling oxygen and PEF treatment parameters, while the migration of additional iron ions should be avoided.

When PEF technology is combined with oxygen in practical production, precise control of the dissolved oxygen levels can be achieved through various means such as low speed, air pressure, and artificial containers. However, studies on how different dissolved oxygen levels affect the effectiveness of PEF treatment still need to be improved. It is hoped that this paper can provide directions for future research and industrial applications.

## Figures and Tables

**Table 1:** Processing parameters and relevant physicochemical indices of strong-flavor baijiu sample.

Factors	CK	S1	S2 (F2)	S3	F1	F3
Electric field strength (kV/cm)	-	15	25	35	25	25
Pulse frequency (Hz)	-	350	350	350	200	500
Alcohol Content (%vol)	67.20 ± 0.70 ^b^	69.47 ± 0.55 ^b^	65.10 ± 1.10 ^a^	67.73 ± 0.95 ^ab^	66.08 ± 1.00 ^a^	66.03 ± 0.15 ^a^
Conductivity (μS/cm)	11.80 ± 0.07 ^a^	13.57 ± 0.80 ^b^	15.10 ± 0.96 ^b^	13.97 ± 0.12 ^b^	17.47 ± 0.74 ^c^	13.90 ± 0.12 ^b^
pH	3.98 ± 0.20 ^a^	3.92 ± 0.05 ^a^	3.94 ± 0.07 ^a^	3.94 ± 0.02 ^a^	4.02 ± 0.08 ^a^	3.94 ± 0.02 ^a^
Dissolve oxygen (mg/L)	7.34 ± 0.14 ^a^	7.34 ± 0.14 ^a^	7.58 ± 0.05 ^b^	7.50 ± 0.09 ^b^	7.42 ± 0.08 ^ab^	7.50 ± 0.09 ^b^
Electrical potential (mV)	175.00 ± 3.34 ^a^	175.83 ± 4.34 ^a^	172.40 ± 2.63 ^a^	177.27 ± 3.68 ^a^	177.27 ± 1.20 ^a^	177.27 ± 3.68 ^a^

^a,b,c^ different superscript letters for the same indices in one line denote significant differences (*p* < 0.05), *p* value of indices of two groups baijiu was assessed by one-way ANOVA.

**Table 2:** Strong-flavor baijiu sample content of various volatile substance categories.

Categories (mg/L)	Esters	Alcohols	Acids	Aromatics	Aldehydes and Ketones
CK	2707.3 ± 238.92 ^a^	124.92 ± 14.8 ^a^	96.11 ± 3.69 ^a^	17.7 ± 9.47 ^a^	10.65 ± 1.26 ^a^
S1	3167.31 ± 363.68 ^a^	200.39 ± 11.57 ^b^	181.32 ± 27.57 ^d^	21.76 ± 0.35 ^b^	12.86 ± 0.19 ^a^
S2 (F2)	2901.81 ± 208.24 ^a^	271.73 ± 44.61 ^c^	132.36 ± 16.36 ^b^	24.37 ± 9.9 ^a^	15.99 ± 0.52 ^b^
S3	2883.73 ± 629.44 ^a^	168.45 ± 6.75 ^b^	155.75 ± 1.81 ^c^	26.7 ± 0.68 ^c^	13.52 ± 1.71 ^a^
F1	3028.13 ± 152.9 ^a^	267.93 ± 23.4 ^c^	137.14 ± 10.3 ^b^	26.02 ± 1.67 ^c^	13.57 ± 1.75 ^a^
F3	3897.49 ± 281.74 ^b^	201.8 ± 22.02 ^b^	181.9 ± 19.2 ^d^	34.09 ± 0.89 ^d^	16.66 ± 1.54 ^b^

^a,b,c,d^ different superscript letters in a list denote significant differences (*p* < 0.05); *p* value of volatile compound categories of seven groups baijiu was assessed by one-way ANOVA. S1–S3 and F1–F3 were the experimental groups, and the electric field strength from S1 to S3 was 15 kV, 25 kV, and 35 kV. The pulse frequency from F1 to F3 was 200 Hz, 350 Hz, and 500 Hz.

## Data Availability

The original contributions presented in the study are included in the article, further inquiries can be directed to the corresponding author.

## Appendix B

**Table A1:** Relative content results of volatile flavor compounds determined by HS-SPME combined with GC-MS.

Compound Name	Blank (mg/L)	S1 (mg/L)	S2 (F2) (mg/L)	S3 (mg/L)	F1 (mg/L)	F3 (mg/L)
3-methylbutyl pentanoate	1.688	2.007	2.239	2.124	2.474	2.316
Ethyl heptanoate	348.671	234.362	263.123	242.753	297.115	264.634
Pentyl Hexanoate	40.843	24.228	24.708	23.626	35.625	29.245
Propyl n-Octanoate	4.186	2.377	2.477	2.725	3.085	2.983
Hexyl hexanoate	193.759	118.620	133.816	130.156	170.265	155.251
Ethyl myristate	23.994	14.210	15.430	13.204	19.178	17.115
Ethyl valerate	69.105	124.348	106.662	127.159	109.528	115.971
Ethyl formate	0.372	0.471	0.507	0.209	0.172	0.619
Ethyl acetate	59.371	112.534	112.723	95.301	90.590	92.946
Ethyl propionate	1.162	1.162	1.162	1.162	-	-
Ethyl isobutyrate	0.423	1.629	1.263	1.996	2.609	2.399
Ethyl butyrate	57.685	103.360	95.648	105.311	94.441	106.623
Ethyl 2-methylbutyrate	0.809	0.997	0.201	1.611	1.369	0.621
Ethyl isovalerate	1.148	4.184	4.307	3.259	2.138	1.352
Isoamyl acetate	3.519	5.322	5.084	6.516	5.488	6.410
Ethyl pyruvate	2.555	2.468	2.201	4.034	2.571	2.518
Ethyl hexanoate	1170.524	1206.133	1251.237	1217.046	1264.031	1261.510
Isoamyl butyrate	4.105	3.635	3.371	3.579	3.424	3.828
Hexyl acetate	7.506	6.819	7.589	8.093	7.306	7.197
Ethyl cyclopropanepropionate	0.830	0.287	0.419	0.626	0.135	-
Ethyl 5-methylhexanoate	4.005	1.536	5.301	5.090	4.654	5.446
Ethyl 3-hexenoate	0.479	1.128	0.991	-	1.055	0.987
Butyl valerate	3.118	1.652	1.795	3.381	0.108	0.360
Propyl hexanoate	35.818	27.427	30.692	27.106	26.679	34.411
Ethyl hexadienoate	1.498	0.666	0.554	1.117	1.663	1.070
Isobutyl hexanoate	9.020	1.268	-	-	8.555	-
Ethyl lactate	9.536	14.838	13.251	10.544	11.834	12.452
Heptyl acetate	0.333	0.189	-	-	-	-
Butyl hexanoate	86.300	69.305	32.877	59.224	67.344	72.534
Ethyl octanoate	539.066	383.160	431.965	445.513	492.647	545.636
Isoamyl hexanoate	60.389	51.464	52.187	49.559	57.250	64.045
Ethyl 7-octenoate	0.353	0.252	0.294	0.091	0.280	0.323
Ethyl nonanoate	28.989	19.477	25.425	21.802	25.755	27.996
Ethyl 2-hydroxy-4-methylpentanoate	2.439	3.925	3.430	3.353	3.288	3.646
Isobutyl heptanoate	0.465	0.770	0.473	0.279	0.912	0.365
Isobutyl octanoate	1.066	0.947	0.953	1.117	1.164	1.225
Ethyl 3-nonenoate	0.677	1.223	1.396	1.647	1.466	1.691
Octyl butyrate	0.373	0.357	0.373	0.367	0.458	0.457
Ethyl decanoate	56.115	33.791	42.831	45.291	47.867	44.538
Isoamyl octanoate	5.173	3.660	4.377	4.622	4.324	5.168
Diethyl succinate	1.593	2.684	2.316	2.126	2.337	2.677
Ethyl 4-decenoate	0.287	0.097	0.095	0.119	-	-
Butyl heptanoate	15.740	4.652	5.758	5.205	5.481	5.481
Ethyl undecanoate	2.149	0.785	1.142	1.349	1.046	1.373
Ethyl 2-decenoate	0.496	0.353	0.370	0.413	0.385	0.458
Ethyl phenylacetate	6.967	9.960	10.421	10.783	11.475	12.405
Hexyl octanoate	18.397	16.506	18.701	14.839	19.805	21.311
Ethyl laurate	24.536	3.556	12.572	21.615	17.119	6.766
Ethyl 3-phenylpropionate	7.525	9.245	11.140	10.896	11.533	11.648
Ethyl tridecanoate	1.067	0.737	0.739	0.723	0.869	0.915
Ethyl 4-phenylbutyrate	-	0.727	0.739	0.757	0.845	0.829
Octyl octanoate	0.654	0.497	0.502	0.477	0.576	0.667
Ethyl pentadecanoate	3.340	2.067	2.177	2.298	1.987	2.599
Methyl palmitate	0.079	0.246	0.232	0.281	0.270	0.315
Ethyl palmitate	156.714	98.682	99.126	114.452	106.419	115.440
Ethyl 9-hexadecenoate	9.645	5.471	6.320	6.188	5.866	6.968
Ethyl 15-methylheptadecanoate	0.629	0.286	0.296	0.223	0.244	0.096
Ethyl oleate	28.083	13.952	15.010	14.786	13.586	14.314
Ethyl linoleate	31.557	15.490	16.625	15.487	14.238	15.593
Dibutyl phthalate	0.224	0.873	1.130	1.568	1.061	1.455
Propyl octanoate	4.186	2.377	2.477	2.725	2.650	3.236
Ethyl undecanoate	2.149	0.785	1.142	1.349	1.046	1.373
Ethyl 2-decenoate	0.496	0.353	0.370	0.413	0.385	0.458
Ethyl phenylacetate	6.967	9.960	10.421	10.783	11.475	12.405
Hexyl octanoate	18.397	16.506	18.701	14.839	19.805	21.311
Ethyl palmitate	156.714	98.682	99.126	114.452	106.419	115.440
Ethyl 9-hexadecenoate	9.645	5.471	6.320	6.188	5.866	6.968
Ethyl oleate	28.083	13.952	15.010	14.786	13.586	14.314
Ethyl linoleate	31.557	15.490	16.625	15.487	14.238	15.593
Butyl valerate	3.118	1.652	1.795	3.381	0.108	0.360
Acetaldehyde	0.327	0.477	0.600	0.714	0.469	0.675
Isovaleraldehyde	3.576	5.371	5.545	6.509	5.626	6.706
Isobutyraldehyde	0.225	0.446	0.447	0.402	0.394	0.470
Nonanal	1.301	0.466	0.266	1.075	1.075	1.075
Butyraldehyde	-	0.157	0.324	0.363	-	-
2-Nonanone	1.324	1.233	1.224	1.559	1.544	1.108
2-Undecanone	0.828	0.512	0.587	0.649	0.682	0.723
2-Pentadecanone	0.720	0.511	0.511	0.477	0.574	0.717
Fitone	0.508	0.314	0.277	0.449	0.221	0.384
2-Nonen-4-one	0.185	-	0.158	-	-	-
Benzeneacetaldehyde, diethyl acetal	0.634	1.556	1.597	1.342	1.446	1.925
Decane, 1,1-diethoxy-	0.005	0.639	0.728	0.648	0.777	0.837
1,1-diethoxynonane	1.933	2.781	3.118	2.759	2.782	3.626
Nonanoic acid	7.760	15.258	21.191	11.021	12.849	25.987
Acetic acid	1.933	2.781	3.118	2.759	3.158	3.980
Isobutyric acid	4.761	3.808	3.681	4.290	4.222	4.638
Butyric acid	1.048	4.010	2.852	-	0.650	3.162
Valeric acid	1.858	2.831	2.417	2.380	2.419	2.566
Hexanoic acid	72.065	141.949	111.350	103.087	102.015	120.088
Heptanoic acid	5.540	8.184	7.818	5.644	6.423	9.758
Octanoic acid	7.760	15.258	21.191	12.086	14.749	27.998
Nonanoic acid	-	0.145	-	0.176	0.239	0.059
3-Hydroxylauric acid	0.036	0.072	0.189	0.134	-	-
α-Linolenic acid	0.479	0.442	0.124	0.478	0.444	0.445
2-Dodecenoic acid	0.474	1.148	1.262	1.555	1.323	1.497
5-Dodecenoic acid	-	0.524	0.534	1.504	1.504	1.504
9-Hexadecenoic acid	-	0.070	0.232	0.263	0.074	0.110
13-Octadecenoic acid	-	0.090	0.174	0.158	0.238	0.363
17-Octadecynoic acid	-	0.393	-	-	-	-
Eicosenoic acid	0.605	0.432	0.333	0.364	0.263	1.144
Hexanoic anhydride	5.516	6.436	7.460	6.499	8.178	7.740
14-Pentadecanoic acid	-	0.188	0.448	0.397	0.452	0.166
Ethanol	103.724	160.902	130.184	234.262	232.228	155.995
Hexanol	19.775	36.768	36.902	36.275	34.207	43.713
Isobutanol	1.103	2.400	1.050	1.197	1.390	1.699
2-Propyl-1-pentanol	-	0.298	0.328	0.211	0.526	0.238
3-Methyl-1-heptanol	-	1.372	3.318	2.759	1.376	-
2-Ethylhexanol	-	-	0.171	0.589	0.302	0.574
Isoamyl alcohol	-	10.298	21.137	6.880	-	-
2-Heptanol	-	0.278	-	-	0.316	-
2-[(Z)-9-Octadecenyloxy]ethanol	0.324	0.089	0.075	0.058	-	0.200
2-Methyl-1-propanol	-	0.204	0.218	-	-	-
Methanol	0.320	0.129	0.317	0.683	0.126	0.696
Propanol	-	0.108	0.355	0.209	0.314	0.154
Butanol	0.167	1.887	0.428	0.988	0.380	1.527
Pentanol	0.431	0.431	0.431	0.431	0.431	0.431
sec-Butanol	-	0.555	0.636	-	0.454	0.645
2-Heptanol	-	0.278	-	-	0.316	-
p-Cresol	0.280	0.290	-	0.210	0.089	0.322
2,4-Di-t-butylphenol	6.512	5.731	16.466	5.701	19.059	19.406
2-(12-Pentadecynyloxy)tetrahydro-2H-pyran	-	0.242	0.783	0.740	0.661	0.958
2-Methylphenol	-	-	-	-	4.428	10.754
2,6-Di-tert-butyl-p-cresol	-	0.122	0.311	0.094	0.097	0.491
Guaiacol	-	0.264	-	0.591	0.212	0.667

**Table A2:** Major relative compounds in strong-flavor baijiu treated with different electric strengths and pulse frequencies of PEF.

Name	Pearson’s r	*p* Value
Octanoic acid	0.2288	0.012
Hexanoic acid	0.1040	0.018
1,1-diethoxynonane	0.2706	0.007
2-(12-Pentadecynyloxy) tetrahydro-2H-pyran	0.1336	0.018
Ethyl caprate	0.1758	0.011
Hexyl hexanoate	0.3733	0.001
Decane, 1,1-diethoxy-	0.2290	0.010
Hexan-1-ol	0.1239	0.038
Hexanoic acid, anhydride	0.2752	0.003
Hexanoic acid,5-methyl-, ethyl ester	0.1240	0.009
1,1-diethoxynonane	0.2484	0.010
Ethyl caprate	0.1332	0.012
Hexyl hexanoate	0.3357	0.003
Decane, 1,1-diethoxy-	0.2026	0.015
Butyl valerate	0.0846	0.048
Hexanoic acid, anhydride	0.2961	0.002

## References

[1] Qiao L., Wang J., Wang R. (2023). A review on flavor of Baijiu and other world-renowned distilled liquors. Food Chem. X.

[2] Jia W., Ma R., Hu L.B., Mo H. (2022). Synergy of physicochemical reactions occurred during aging for harmonizing and improving flavor. Food Chem. X.

[3] Shui Z., Zhao J., Li Y. (2025). Fast identification of Baijius based on organic acid response colorimetric sensor array. J. Food Compos. Anal..

[4] Sun Q., Yang R., Wu L. (2015). High-voltage pulsed electric field has sterilization and aging effects on fermented orange vinegar. Sci. Technol. Food Ind..

[5] Jia W., Fan Z., Du A. (2021). Untargeted foodomics reveals molecular mechanism of magnetic field effect on Feng-flavor Baijiu ageing. Food Res. Int..

[6] Rosellini T.K., Aline A., Alessandro N. (2022). Current Technologies to Accelerate the Aging Process of Alcoholic Beverages: A Review. Beverages.

[7] Parisa M.G., Shima J., Jia G. (2022). Ozone in wineries and wine processing: A review of the benefits, application, and perspectives. Compr. Rev. Food Sci. Food Saf..

[8] Andreou V., Giannoglou M., Xanthou M.Z., Metafa M., Katsaros G. (2023). Aging acceleration of balsamic vinegar applying micro-oxygenation technique. Food Chem..

[9] Puértolas E., López N., Condón S. (2010). Potential applications of PEF to improve red wine quality. Trends Food Sci. Technol..

[10] Bai C.X., Yang Y. (2018). Pulsed electric fields as an alternative to thermal processing for preservation of nutritive and physicochemical properties of beverages: A review. J. Food Process Eng..

[11] Zi L., Pang Z., Wen M. (2023). Pulsed electric field processing of green tea-infused chardonnay wine: Effects on physicochemical properties, antioxidant activities, phenolic and volatile compounds. Food Biosci..

[12] Toulaki A.K., Bozinou E., Athanasiadis V. (2023). Accelerating Xinomavro Red Wine Flavor Aging Using a Pulsed Electric Field and Various Wood Chips. Appl. Sci..

[13] Zhang C., Zhao W., Yan W. (2021). Effect of pulsed electric field pretreatment on oil content of potato chips. LWT.

[14] Lin Z., Zeng X., Yu S. (2011). Enhancement of Ethanol–Acetic Acid Esterification Under Room Temperature and Non-catalytic Condition via Pulsed Electric Field Application. Food Bioprocess Technol..

[15] Yu Q., Zeng X. (2013). Effect of PEF treatments on enhancing the chelation reaction between glycine and copper sulfate. Food Ferment. Ind..

[16] Del A.S., Pando M., Nevares V. (2014). Investigation and correction of the interference of ethanol, sugar and phenols on dissolved oxygen measurement in wine. Anal. Chim. Acta.

[17] Zhang Q., Whang Z., Xiong A. (2021). Elucidating oxidation-based flavour formation mechanism in the aging process of Chinese distilled spirits by electrochemistry and UPLC-Q-Orbitrap-MS/MS. Food Chem..

[18] Elias R.J., Mogens L.A., Leif H.S. (2009). Identification of Free Radical Intermediates in Oxidized Wine Using Electron Paramagnetic Resonance Spin Trapping. J. Agric. Food Chem..

[19] Qing Z., Yuan S., Xue F. (2015). Free radical generation induced by ultrasound in red wine and model wine: An EPR spin-trapping study. Ultrason. Sonochem..

[20] Xie F., Mao H., Lin C., Feng Y., Stoddart J.F., Young R.M., Wasielewski M.R. (2023). Quantum Sensing of Electric Fields Using Spin-Correlated Radical Ion Pairs. J. Am. Chem. Soc..

[21] Deng Y., Xiong A., Zhao K. (2020). Mechanisms of the regulation of ester balance between oxidation and esterification in aged Baijiu. Sci. Rep..

[22] Pérez M.S., Martínez G.A., Bueno H.M. (2023). Kinetics of oxygen consumption, a key factor in the changes of young wines composition. LWT.

[23] Zhao W., Yang R., Gu Y., Tang Y., Li C. (2014). Assessment of pulsed electric fields induced cellular damage in Saccharomyces cerevisiae: Change in performance of mitochondria and cellular enzymes. LWT—Food Sci. Technol..

[24] He Y., Tang K., Yu X. (2022). Identification of Compounds Contributing to Trigeminal Pungency of Baijiu by Sensory Evaluation, Quantitative Measurements, Correlation Analysis, and Sensory Verification Testing. J. Agric. food Chem..

[25] (2012). Sensory analysis-General guidelines for the selection, training and monitoring of selected assessors and expert sensory assessors.

[26] Sun X., Qian Q., Xiong Y. (2022). Characterization of the key aroma compounds in aged Chinese Xiaoqu Baijiu by means of the sensomics approach. Food Chem..

[27] Liu Q., Zhang X., Zheng L. (2023). Machine learning based age-authentication assisted by chemo-kinetics: Case study of strong-flavor Chinese Baijiu. Food Res. Int..

[28] (2018). Baijiu Analyticl Methods.

[29] Zhong P., Lin Z., Liu Z., Kong L. (2005). Source, species and determination method of reactive oxygen species in water environment. Ecol. Sci..

[30] Wang M.S., Wang S.N. (2021). Study on the Variable-Frequency Electric Field Assisted Aging Mechanism of Liquor. Nucl. Ind. Inst. Chem. Eng..

[31] Everendilek G.A. (2022). Pulsed Electric Field Processing of Red Wine: Effect on Wine Quality and Microbial Inactivation. Bverages.

[32] Wei Z., Yang X., Ru D. (2023). An electric-field instrument for accelerated aging to improve flavor of Chinese Baijiu. LWT-Food Sci. Technol..

[33] Qing R., Liu X.L. (2024). Influence on the volatilization of ethyl esters: Nonnegligible role of long-chain fatty acids on Baijiu flavor via intermolecular interaction. Food Chem..

[34] Feng Y., Yang T., Zhang Y., Zhang A., Gai L., Niu D. (2022). Potential applications of pulsed electric field in the fermented wine industry. Front. Nutr..

[35] Zhang Q.A., Zheng H., Lin J. (2023). The state-of-the-art research of the application of ultrasound to winemaking: A critical review. Ultrason. Sonochem..

[36] Martin J.F.G., Sun D.W. (2013). Ultrasound and electric fields as novel techniques for assisting the wine ageing process: The state of the art research. Trends in Food Sci. Technol..

[37] Zhu L., Wang X., Song X. (2020). Evolution of the key odorants and aroma profiles in traditional Laowuzeng baijiu during its one-year ageing. Food Chem..

[38] Qu J., Chen X., Wang X. (2024). Esters and higher alcohols regulation to enhance wine fruity aroma based on oxidation-reduction potential. LWT.

[39] Comuzzo P., Marconi M., Zanella G. (2018). Pulsed electric field processing of white grapes (cv. Garganega): Effects on wine composition and volatile compounds. Food Chem..

[40] He J., Chen Q., Jia X. (2022). The effects of gamma irradiation and natural aging on the composition of Nongxiangxing baijiu. J. Food Process. Preserv..

[41] Cheng Z., Xiao L., Arshad R.N. (2023). Pulsed electric field as a promising technology for solid foods processing: A review. Food Chem..

[42] Zheng Q., Hu Y., Xiong A., Su Y., Wang Z., Zhao K., Yu Y. (2021). Elucidating metal ion-regulated flavour formation mechanism in the aging process of Chinese distilled spirits (Baijiu) by electrochemistry, ICP-MS/OES, and UPLC-Q-Orbitrap-MS/MS. Food Funct..

[43] Jang M.L., Hu X.J., Lei Y. (2022). Research progress of liquor aging. Chin. Brew..

[44] Wei L., Hu J., Pan C. (2023). Effects of different storage containers on the flavor characteristics of Jiangxiangxing baijiu. Food Res. Int..

[45] Sha S., Chen S., Qian M. (2017). Characterization of the typical potent odorants in Chinese roasted sesame-like flavor type liquor by headspace solid phase microextraction-aroma extract dilution analysis, with special emphasis on sulfur-containing odorants. J. Agric. Food Chem..

[46] Yan Y., Chen S., Nie Y. (2020). Characterization of volatile sulfur compounds in soy sauce aroma type Baijiu and changes during fermentation by GC × GC-TOFMS, organoleptic impact evaluation, and multivariate data analysis. Food Res. Int..

[47] Huang Z.J., Zeng Y.H., Liu W.H., Wang S.T. (2020). Effects of metals released in strong-flavor baijiu on the evolution of aroma compounds during storage. Food Sci. Nutr..

[48] Liu X., Millero F.J. (1999). The solubility of iron hydroxide in sodium chloride solutions. Geochim. Cosmochim. Acta.

[49] Thomas B.N. (1963). Chemistry of Iron in Natural Water. United States Government Printing Office. https://pubs.usgs.gov/wsp/1459a/report.pdf.

[50] Modesti M., Macaluso M., Taglieri I. (2021). Ozone and Bioactive Compounds in Grapes and Wine. Foods.

